# Immunization review meetings: “Low Hanging Fruit” for capacity building and data quality improvement?

**DOI:** 10.11604/pamj.supp.2017.27.3.11516

**Published:** 2017-06-22

**Authors:** Lora Shimp, Nassor Mohammed, Lisa Oot, Evans Mokaya, Timothy Kiyemba, Gerald Ssekitto, Adriana Alminana

**Affiliations:** 1 John Snow, Inc, USA; 2 Maternal and Child Survival Program,Tanzania; 3 JSI Research and Training Institute, Inc; 4 Maternal and Child Survival Program, Kenya; 5 Maternal and Child Survival Program, Uganda

**Keywords:** Review meeting, routine immunization program strengthening, performance improvement, best practices, lessons learned, peer learning, peer exchange, adult learning, data quality and use

## Abstract

**Introduction:**

Although systematic program review meetings are common practice in many health and immunization programs, there is little documentation on their implementation and role. Adult education principles espouse opportunities for peer exchange to build capacity and cross-learning, for which review meetings have been a forum utilized in immunization programs for many years. This study describes the process and use of review meetings to build immunization technical capacity in four African countries since 2011.

**Methods:**

A longitudinal case study providing retrospective descriptive analysis and qualitative data collected on immunization program implementation and review meetings conducted within the years of 2011-2016 with district and facility health staff and technical partners from Ethiopia, Kenya, Tanzania and Uganda.

**Results:**

Based on summarized findings and analyses from over 200 review meetings conducted in the four countries within the time period of 2011-2016, these meetings have been shown to be effective tools for improving immunization program performance and the capacity of health staff.

**Conclusion:**

Review meetings (ideally conducted quarterly) provide health workers with beneficial and low cost opportunities for adult learning, including building skills in data analysis and review, which can be sustained at district and health facility levels. In combination with other performance improvement approaches implemented and supported in countries (such as supportive supervision, training, and on-the-job learning and assessment), review meetings can also contribute to achievement of immunization and health outcomes.

## Introduction

Regular review of immunization data is essential not only for countries to monitor and improve their immunization program performance, but also to strengthen accountability, especially for countries whose programs are mainly funded by large donor initiatives such as Gavi, the Vaccine Alliance [1]. However, the question of who reviews immunization data is also important, as performance estimates can be subject to bias and over reporting when sent to higher levels [2]. The systematic review of performance by peers can be one strategy to mitigate incentives to over report and also to improve data quality. This experience exchange has been found to be a highly effective method of learning. Research has shown that people learn well from others who are at an equivalent level to them, as they have similar challenges and can share their knowledge, ideas, and experiences with each other [3]. Thus, many health programs-including immunization-have incorporated regular (e.g. quarterly or biannual) review meetings with participation by peers as a means for knowledge sharing and performance improvement. However, there is little documentation on the quality of these review meetings, notably on their role vis-à-vis the various other reporting and feedback processes and tools used in immunization programs for improving services, data monitoring and use, and towards promoting a culture of utilizing data for decision-making.

Key World Health Organization (WHO) Expanded Program on Immunization (EPI) documents, such as training documents for Mid-Level Managers (MLM) as well as the WHO Africa Region's Reaching Every District (RED) Approach field guide, promote regular review meetings as an opportunity to discuss data trends, achievements, and promote peer exchange and problem-solving [4,5]. The RED Approach field guide lists the percentage of districts conducting at least one review meeting per quarter as a core indicator within the RED strategy.

Though common practice in many health programs, review meetings have generally had limited documentation in published literature. In Zambia, review meetings centered on maternal mortality were viewed as “an important tool for improving-services,” providing a data-driven forum to address quality-of-care issues, train and educate staff, and create a shared sense of understanding and purpose for attendees [6]. Similarly in Mozambique, review meetings were used as a means to improving malaria service delivery through peer review and presentation of data, with a focus on participants providing constructive critiques and generating recommendations for performance improvement [7]. Review meetings for routine immunization (RI) programs have also been hailed as an important tool to improve performance if they are used to document and discuss lessons learned among peers and maintain a focus on problem solving [8, 9]; if instead review meetings are based on criticism, as shown in Mozambique, it does not contribute to the adequate support mechanisms needed to improve RI system performance [10].

This case study describes the processes and use of review meetings, notably at district level, in Ethiopia, Kenya, Tanzania, and Uganda (within the years of 2011-2016) as complementary opportunities with training and other approaches for adult learning and capacity building in immunization program implementation. These experiences have contributed to improvements in immunization data quality through empowering health workers to understand, use and appreciate the data that they are collecting, learn from one another, and apply the learning and findings in their day-to-day activities.

## Methods

This article provides a retrospective, longitudinal descriptive analysis. The qualitative data and findings collected on immunization program implementation and assessments come from review meetings conducted within the years of 2011-2016 with district and health facility health staff from approximately 100 districts in four countries: Ethiopia, Kenya, Tanzania and Uganda. The districts in which the review meetings were conducted were determined using varying criteria agreed upon by the respective Ministry of Health EPI programs and technical partners in each country, with district selection primarily based on routine immunization coverage trends and/or those districts with high numbers of un/under-vaccinated children (from surveyed and/or routine administrative coverage data) in previous years' birth cohorts. Rapid immunization assessments in Kenya and Tanzania, using a “Rapid Assessment of Immunization in Districts” (RAPID) approach [11] adapted from India revealed significant data quality gaps contributing to low performance in districts. Some of the gaps included: reported coverage of below 80% or over 100% for several months; disparities in coverage among vaccines that are to be administered in the same visit; negative drop-out rates for several months; coverage figures that do not align between separate data reports in which the coverage numbers should be the same; and fully immunized child (FIC) coverage higher than coverage figures for measles, OPV, and pentavalent vaccines. Worryingly, the health workers, and in some instances their supervisors, did not identify these errors nor realize that there were problems with the data prior to these assessments. The immunization review meetings discussed in this article (generally held quarterly and referred to subsequently as QRMs) are part of a combination of approaches for capacity building and performance improvement [12] already in place in the countries and therefore not separate initiatives or case-control studies. These QRM examples come from district level and involve Ministry of Health (MOH) colleagues from health facilities, districts, and in some cases regional and national levels. As the QRMs are part of existing MOH immunization program activities and had some co-facilitation and support by in-country technical partners external to the MOH, the methodologies and timeframes discussed differ between countries. This summary therefore focuses on the commonalities and best practices found between the QRMs and highlights specificities and learning from the respective countries' planning and implementation.

## Results

**Figure 1:**
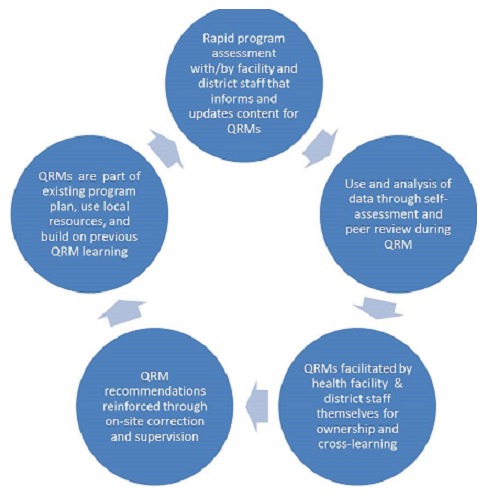
Key elements of a review meeting

The QRM activities in each country (Ethiopia, Kenya, Tanzania and Uganda) have been tailored to fit existing health and immunization program structures and capacity, so as to minimize cost and/or to link with other supervision, RED approaches and methods for data quality assessment and use that are already in place. Although the review meetings were budgeted and costed as part of in-country immunization program plans, formal QRM cost analyses were not conducted in the countries included in this study. Key elements that each country's QRMs include are noted below (Figure 1, review meeting key elements) and described subsequently in the county summaries: the content and structure of QRMs are based on initial rapid assessments of the routine immunization program conducted with district and facility staff at the facilities themselves, sometimes incorporating on-site corrections.

In Kenya and Tanzania, this was part of the RAPID approach noted previously; whereas in Ethiopia and Uganda, this was a component within a REC-QI (Reach Every Child-Quality Improvement) [13] framework, subsequently described in those specific country summaries. Locally generated immunization administrative data are shared by health staff and discussed for self-analysis of performance and achievement. Comparison and feedback on the data and findings are provided through a peer review process between users and colleagues that incorporates adult learning principles. The findings, summarized analyses, recommendations and feedback provided during the QRM are addressed by the facility and district level staff on-the-job between QRMs, incorporating on-site correction and/or through supervision visits or follow-up communications. Each QRM builds on the previous QRM recommendations and discussions to reinforce and supplement learning and practices, with data visualization to see trends, progress and constraints. The QRMs are part of the immunization program annual planning and utilize local human and financial resources, although some initial supplemental funding may be used to help institute the process or to enable immunization-focused sessions or days (e.g. in the case of QRMs that integrate several health initiatives).

**Kenya:** In 21 subcounties/districts beginning in 2011, rapid immunization assessments were conducted with district and facility staff, with some limited initial orientation provided by Kenyan immunization experts working with the Maternal and Child Integrated Program (MCHIP) [14] and its follow-on Maternal and Child Survival Program (MCSP) [15]. The assessments found poor data quality and lack of data use to be significant contributors to low performance in these districts. Health workers (and in some instances their immediate supervisors) had not previously identified problems with data or performance, in large part because they had not received in-service EPI training or updates for several years. These assessments have been used to help inform the content of the QRMs from 2011 through 2016. The QRMs have been part of implementation of the RED approach and periodic data quality self-assessment (DQSA) for health workers to review their own data and engage with communities for monitoring immunization services. Prior to a QRM being conducted, an average of 5-10 health facilities are visited by the district/county team for supportive supervision, with a DQSA also conducted in 3-5 of these facilities. At the QRM, each facility is required to bring hard copies of their monthly immunization summary reports to compare with the district and DHIS data. The first part of the QRM involves immunization data verification using the facility immunization tally sheets. After the data verification exercise, each health worker is provided an opportunity during the QRM to interpret their facility immunization performance and identify access and/or utilization problems. The next part of the QRM involves selected facilities (e.g. most improved, lower and higher performing facilities) presenting their analysis to the other health workers for peer review and feedback, with special focus on the drivers for their performance (see Annex 1: Quotes from health workers in Kenya QRMs).

Annex 1 **Quotes from health workers in Kenya QRMs in August 2016:**

“*I did not know that data could reveal the challenges I face in my facility.*”

“*Next time I must present in this meeting, so I have to improve my documents.*”

“*At least I can now speak the data language: poor utilization, good access*”

“*I have never appreciated data like I have in this meeting…*”

“*[In this meeting], you have removed the cobwebs in my eyes as far as immunization data is concerned.*”

At the end of the QRM, the district and facility teams then review and discuss the findings of supportive supervision, DQSA, the harmonized data and the selected facilities' performance presentations. These QRM activities have provided an opportunity for health workers to share best practices and challenges, address contentious issues, and identify actions points and recommendations for follow-up within the facilities and before the next review meeting. After a few rounds of review meetings, health workers improved their interpretation of immunization data and correctly completed immunization monitoring charts. The discussions in review meetings have been strengthened with active participation from even the initially more timid health workers. As shown in this example from Nyakach sub-county (Table 1), as review meetings continued over time, there were declines in: the number of facilities that reported data with disparities in coverage among vaccines given in the same visit, Fully Immunized Child coverage reported to be higher than measles or pentavalent 3 coverage, and negative drop-out rates for more than 6 months.

**Table 1:** Number of health facilities in Nyakach sub-county with data disparities over the course of five QRMs

Nyakach sub-county (n=29 facilities)					
Data gaps	1st review meeting	2nd review meeting	3rd review meeting	4th review meeting	5th review meeting
Disparities between OPV, Penta and PCV coverage	18	18	11	12	2
FIC > Measles coverage	26	27	13	10	10
FIC > Penta 3 coverage	8	5	6	2	0
Negative drop out	9	8	4	3	3

Source: routine administrative immunization coverage data for Nyakach sub-county, January 2015 – June 2016

**Figure 2:**
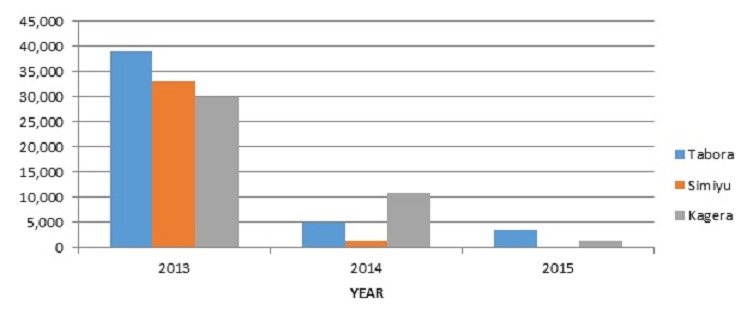
Number of under-vaccinated children (0-11 months, not received Penta 3) by year, in 3 selected regions of Tanzania

Tanzania: Based on immunization administrative coverage trend analyses conducted in Tanzania in 2013, the regions of Tabora, Simiyu and Kagera were identified as having approximately 40% of the under-vaccinated in the country. RAPID assessments in 13 districts in these regions were conducted in 2013-2014 by the regional and district health and immunization teams with support from MCHIP to identify challenges and needs to improve their immunization performance. Several interventions, including review meetings, were proposed and implemented to improve planning and reliable immunization budgeting and funding, the capacity of health workers, and the operationalization of Reaching Every Community (REC). From mid-2014 through August 2016, four review meetings per district have been conducted, involving heath care workers from all immunizing health facilities in the districts. The average cost for conducting a review meeting with approximately 40 participants is US$5,000. The meetings discussed performance of all health facilities and program management needs/constraints, analyzed immunization data and quality issues, and enabled peer exchange between better and poorer performing facilities. As some of the recommendations from the meetings required management and support from other partners, they were followed by Primary Health Care Committee meetings (chaired by District Commissioners) involving local government, community and religious leaders. As part of REC, these meetings are also linked with supervision, facility visits/exchange, and on-site corrections, as possible. These efforts are considered to have contributed to improved immunization program performance and reductions in the trends of under-vaccinated in these regions, as shown in Figure 2.

**Ethiopia:** integrated QRMs are held at the woreda (district) level, with woreda, health center, and health post staff together identifying and sharing potential best practices for routine immunization (RI) and other health areas. Each woreda throughout the country is expected by the Federal Ministry of Health to budget and conduct QRMs; although the implementation of these meetings varies widely by woreda, with some supported by partner organizations. Building onto this existing platform, starting in April 2013 and through December 2016, Universal Immunization through Improving Family Health Services (UI-FHS) [16] has provided technical support to over 50 woredas for QRMs and has funded over 100 QRMs (approximately 2 QRMs in each woreda it supports). The financial support was estimated at approximately $4500 USD for each QRM (to support 50 health staff and meeting space for three days). These meetings serve as an opportunity to review and analyze RI data, share best practices, and promote peer to peer learning. Woreda Health Teams (WoHO) organize and facilitate the QRMs, with some limited assistance from UI-FHS to organize and prepare presentations for the meeting. After the initial meeting, the WoHO designs, facilitates and funds subsequent QRMs using a framework which includes RED categorization and quality improvement tools such as Plan-Do-Study-Act (PDSA) cycles [17]. These are used for problem solving of management and service delivery issues related to routine immunization to show health center and health post performance at a disaggregated level. During the QRM, the process of one PDSA cycle from health post, health center or WoHO level is presented and results and changes analyzed and discussed. The meeting also reviews the woreda immunization microplan and emphasizes the importance of integrated supportive supervision. The tools and methods used for RI are now also being used for other health services during QRMs (e.g. antenatal care, family planning, water and sanitation, HIV prevention). QRM summary reports (including participants, main points discussed, participant comments and recommendations) are provided for each meeting and compared with target plans, with two to four QRMs conducted per year based on woreda resources.

**Uganda:** since 2013, promotion of QRMs with health sub-district (HSD) and District Health Management Teams (DHMTs) has been a focus to improve health performance, including for routine immunization. In fifteen districts supported under MCHIP, MCSP and the Stronger Systems for Routine Immunization project (SS4RI) within the period of January 2013-December 2016, a total of 59 QRMs (34 DHMT and 25 HSD levels) have been conducted, with some technical and financial (e.g. US$2300-5000) support from these various projects. These meetings provide an opportunity for district, HSD and facility staff to analyze, use and discuss indicators jointly with non-traditional stakeholders at the respective levels on how to improve and sustain performance. Additionally, the participation of non-health worker stakeholders (i.e. civic, political, religious leaders and civil society organizations) brings in other views on the causes and possible solutions to RI challenges. These leaders are also responsible for allocation of resources in the districts, so the QRMs contribute to their appreciation of resource needs. During a QRM week in a district, three types of meetings are held:

**A preparatory/planning meeting that targets the DHMT and HSDs to:** review the agenda, confirm the targeted participants for the different meetings and align all the necessary logistics, form teams to support HSD QRMs, and agree on the schedule for HSD and DHMT QRMs and technical materials;

**The HSD QRMs are held at the HSD headquarters on days suggested during the DHMT planning meeting:** each HSD holds its QRM separately, with health facility staff, and sub-county political, civil and religious leaders jointly analyzing their performance and developing strategies for improvement using the WHO RED categorization tool. During these meetings, health facilities also present PDSA cycles and results for inter-facility knowledge sharing, adjustment of actions that are not working, and adaptation of learning and best practices.

**The DHMT and district civil, political and religious leaders meet with HSD in-charges to present summary resolutions from HSD QRM meetings:** obtain buy in and support, and generate action points at district level. The cascading from HSD QRMs to DHMT meetings facilitates timely communication and discussion of issues affecting RI identified by the HSDs to the district leadership (DHMT & political and civic leaders). These QRMs have engaged DHMTs and HSDs to take the lead from the onset in reviewing their own performance, including exploring avenues together for sustainability and funding of QRMs. It is critical to maintain attendance of key staff (e.g. District Health Officer, Assistant Chief Administrative Officer, District Secretary for Health, immunization focal point persons, Biostatisticians and other health team members) to assist with follow-up on key issues and to address other health service area linkages. An example of QRM success in addressing challenges facing RI is the case of Nyamiryango Health Center II in Kabale district, which had not vaccinated a single child in 6 months despite having all necessary resources to vaccinate children [18]. The health facility was identified through data analysis (using the RED categorization tool), discussed during the QRM and the district chairperson took interest to resolve the challenge. As a result, Nyamiryango health facility has recorded better performance, including conducting regular static and outreach sessions according to plan and increasing the number of children vaccinated (for example, from 0 to 79 and then to 121 children in two subsequent months, with continued improvement thereafter).

## Discussion

Some of the challenges and needs shared and extensively discussed during the QRMs held in each of these countries included:

- Inadequate use of the child/immunization registers for recording children and lack of triangulation of data between different sources (e.g. registers, child health/immunization cards, immunization tally sheets, monthly immunization summaries, immunization coverage monitoring charts, etc). This has made it difficult for facilities, health staff and mobilizers to track routine immunization defaulters and for health workers to reliably verify that children have been vaccinated. Resolutions discussed have included DHMTs sending circulars or bulletins to health facilities encouraging them to use the child immunization register as a primary RI data collection tool at sessions and to incorporate DQSA into supervision and routine monitoring to improve data quality and use.
- Adapting the use of RAPID and PDSA approaches with all health facilities to improve their use of data for decision making and to strengthen service delivery. These approaches can also assist health teams to identify and implement innovations for improving REC within their facilities and also to share lessons learned (e.g. in future QRMs) with health facilities still facing challenges.
- Ensuring sustainability of QRMs through local resources. In all four countries, the QRMs are linked with government budgets and annual plans. Senior, district and health facility teams have planned and advocated for their attendance at QRMs and have included these in their annual health planning and financial cycles.
- Documenting the QRM discussions through meeting minutes that are shared with participants, supervisors and health teams to help facilitate implementation of recommendations and as a record for review and future follow-up in subsequent field visits and meetings.

## Conclusion

Based on the examples from these countries, review meetings (ideally held at least quarterly and which are already part of-or can be further integrated with - the existing health and immunization planning and budgeting) can be effective tools for improving immunization program performance and the capacity of health staff. Review meetings have the following benefits: Provide an opportunity for health workers to analyze, appreciate, and use the data that they generate themselves to make programmatic improvements and strengthen their capacity; Foster a culture of using data for decision making, regular performance monitoring, and self-assessment; Enable sharing of best practices, lessons learned, peer review and benchmarking using adult learning methods and principles; Serve as an opportunity to also update district teams and health workers on latest technical information and to provide feedback and dialogue on results and indicators; Contribute to program cost-benefits, when based on advance situational assessments and linked/harmonized with existing planning and performance improvement approaches to minimize budgets and maximize use of human and financial resources. (Once the content and quality of the review meetings are established, the time needed may be able to be streamlined or reduced, which can also have cost savings).

**Recommendations**

**Incorporate QRMs as part of a comprehensive health performance improvement strategy that is:** based on situational analyses with the participants of the QRMs, utilizes adult learning principles and methods, and leverages existing human and financial resources.

**Involve participants as co-facilitators:** although some initial external facilitation may be needed as the QRMs are being redesigned/integrated into program implementation, the use of participants as facilitators can minimize the need for and cost of external or higher level facilitation.

**Analyze and estimate costs for QRMs based on local realities, with QRMs planned, budgeted and funded as part of annual immunization and/or health plans:** although the QRM cost averages were between US$2300-5000 in these countries, the standard costs for a review meeting will need to consider country specifics and subnational variations in numbers of participants, transport distances, and other factors.

**Include and/or share review meeting plans, summaries/notes and recommendations with local partners and stakeholders:** to assist in advocacy for their involvement and buy-in as well as for sustainability of achievements.

### What is known about this topic

- As part of health and immunization programs in most African countries, periodic program reviews (e.g. annually, semi-annually, quarterly) are held to review key indicators, discuss program status and needs, and consolidate reporting;
- Systematic review meetings on health program performance are important for information-sharing and as a complement or alternative to more formal classroom or facility/center-based training;
- Research has shown that peers can learn well from one another, share ideas and build knowledge and skills through adult learning methods like review meetings.

### What this study adds

- Although immunization training and supervision have been implemented for decades, there is little documentation on the use, benefit and potential impact of review meetings and peer learning, which this study provides through four country experiences;
- The processes and use of program review meetings, notably at district levels (as documented in the countries in this study), can potentially be modeled or adapted by other countries;
- These immunization review meeting examples demonstrate the contribution of adult learning and peer exchange to strengthening immunization and health services and indicators, as relatively easy and affordable approaches for capacity building and health staff skills development.

## Competing interests

The authors declare no competing interests.
